# High prevalence of anal HPV16 infection among HIV-negative men who have sex with men and transgender women using pre-exposure prophylaxis: a single-centre retrospective cross-sectional study

**DOI:** 10.1016/j.nmni.2026.101758

**Published:** 2026-04-27

**Authors:** Marion de Quillacq, Maxime Bonjour, Sarah Soueges, Florent Valour, Anne-Sophie Batalla, Florence Ader, Hélène Lardot, Anne Conrad, Sylvie Radenne, Matthieu Godinot

**Affiliations:** a Hospices Civils de Lyon, Département des Maladies infectieuses et tropicales, F-69000, Lyon, France; b Hospices Civils de Lyon, Pôle de Santé Publique, F-69000, Lyon, France; c Inserm 1111, Centre International de Recherche en Infectiologie (CIRI), Université Claude Bernard Lyon 1, CNRS UMR5308, École Normale Supérieure de Lyon, Univ Lyon, F-69000, Lyon, France; d Hospices Civils de Lyon, Centre Gratuit d’Information, de Dépistage et de Diagnostic (CeGIDD), Hôpital Edouard Herriot, F-69000, Lyon, France; e Hospices Civils de Lyon, Centre Gratuit d’Information, de Dépistage et de Diagnostic (CeGIDD), Hôpital de la Croix-Rousse, F-69000, Lyon, France; f Hospices Civils de Lyon, Service d’Hépato-gastroentérologie, F-69000, Lyon, France

**Keywords:** Anal human papillomavirus, Pre-exposure prophylaxis, Anal cytology, Men who have sex with men, HPV16, Sexually transmitted infections

## Abstract

**Background:**

Anal squamous cell carcinoma is a preventable digestive malignancy driven by persistent infection with high-risk human papillomavirus (HPV), particularly HPV16. Current anal cancer screening strategies primarily target HIV-positive populations and may not reflect evolving risk profiles in HIV-negative men who have sex with men (MSM) using pre-exposure prophylaxis (PrEP).

**Methods:**

We conducted a retrospective cross-sectional study in HIV-negative MSM and transgender women (TGW) using PrEP who underwent routine proctology consultations. Anal samples were collected for cytology and high-risk HPV DNA testing, and targeted biopsies were performed when lesions were identified on standard anoscopy. We estimated the prevalence of anal HPV16 infection, identified associated behavioral and clinical factors, and assessed the prevalence of cytological and histological anal abnormalities.

**Results:**

Among 458 participants with interpretable HPV testing, anal HPV16 infection was detected in 32.5%. HPV16 positivity was independently associated with chemsex practices (OR 1.76; 95%CI1.14–2.71) and a history of rectal bacterial sexually transmitted infections since PrEP initiation (OR 1.64; 95% CI, 1.10–2.47). Cytological abnormalities were observed in 22.9% of participants and were more frequent in those with HPV16 infection (31.1% vs. 19.1%; OR 1.9; 95%CI 1.18-3.05; p = 0.008). Histological assessment was available for 92 individuals; 18.6% had low-grade lesions and 1.3% had high-grade lesions. Overall, HPV-related histological lesions were present in 19.9% of participants.

**Conclusions:**

HIV-negative MSM and TGW using PrEP exhibit a high burden of anal HPV16 infection and cytological abnormalities. These findings suggest that selected high-risk PrEP users may benefit from targeted anal cancer screening strategies.

## Highlights

• High anal HPV16 prevalence was observed in HIV-negative PrEP users.
• HPV16 prevalence in PrEP users approached levels reported in HIV-positive MSM.
• Chemsex and recent bacterial STIs were independently associated with HPV16 infection.
• Anal cytological abnormalities were frequent and associated with HPV16 infection.
• Findings support extending anal cancer screening to high-risk PrEP users.

## 1 Introduction

Anal squamous cell carcinoma (ASCC) is a rare but increasingly prevalent malignancy [1,2], with the highest burden observed in men who have sex with men (MSM) living with HIV [3]. Persistent infection with high-risk human papillomavirus (HR-HPV), particularly genotype 16 (HPV16), is the main etiological factor driving high-grade squamous intraepithelial lesions (HSIL) and ASCC [4,5].

Over the past two decades, widespread use of highly active antiretroviral therapy, “Treatment as Prevention” (TasP), and, since 2016, freely available pre-exposure prophylaxis (PrEP) have markedly reshaped HIV incidence and sexual behaviours in MSM [6,7]. PrEP use has been associated with an increase in condomless anal intercourses, higher partner turnover, and elevated rates of bacterial sexually-transmitted infections (STIs), including *Chlamydia trachomatis* (CT) and *Neisseria gonorrhoeae* (NG) [8,9]. The rise of geolocated dating apps and recreational drug use during sexual activity (“chemsex”) may further amplify these trends [10,11]. Altogether, bacterial STIs and behavioural shifts could promote anal mucosal inflammation and facilitate HPV acquisition and persistence [12].

A landmark meta-analysis data indicate ASCC incidence rates exceeding 80 per 100 000 person-years in HIV-positive MSM versus 19 per 100 000 in HIV-negative MSM [3]. However, these estimates relied on pre-PrEP studies [13], and may not reflect current epidemiological and behavioural realities. Most prior studies assessed HPV prevalence near PrEP initiation, potentially underestimating long-term infection risk [[14], [15], [16]]. Moreover, data on HPV16 prevalence and its association with cytological or histological anal abnormalities in MSM and transgender women using PrEP remain scarce [[14], [15], [16]].

The primary objective of this study was to estimate anal HPV16 prevalence in HIV-negative MSM and transgender women on PreP. Secondary objectives were: 1) to identify factors associated with HPV16 infection; and 2) to assess the prevalence of cytological or histological anal abnormalities and their association with HPV16, smoking, and HPV vaccination.

## 2 Material and methods

**Study Design and Participants**. This single-centre, retrospective, observational cross-sectional study was conducted in the *Hospices Civils de Lyon* (HCL, Lyon, France). All first anal samples collected between 16th May 2019 and 27th June 2024 from individuals assigned male at birth, aged ≥18 years, receiving PrEP, and undergoing routine proctology consultations as part of local HIV PrEP follow-up were included. Only the first sample was considered for individuals with multiple visits. Patients referred for symptomatic evaluation unrelated to systematic screening were excluded. At the time of the study, standard practice included cytology for all individuals and histology when indicated. HPV testing using PCR was performed for research purposes; only interpretable results were included in the analysis.

**Study endpoints and data collection.** The primary endpoint was the prevalence of anal HPV16 infection. Secondary endpoints included: i) determinants of HPV16 infection, comprising history of anal bacterial STIs, chemsex, use of novel psychoactive substance, smoking (current or past), age, duration of PrEP use, HPV vaccination and urban residence. Urban residence was defined as living in a municipality with ≥2000 inhabitants, and rural residence as <2000 inhabitants; and ii) prevalence of anal cytological and histological abnormalities, and their association with HPV16, smoking and HPV vaccination status. Corresponding demographic, clinical, and behavioural data were retrieved from the Nadis® electronic medical record. History of bacterial STIs within the past 12 months or since PrEP initiation were collected, including CT and NG infections only. Syphilis and anal HSV infections were excluded, as serology cannot identify asymptomatic intra-anal lesions and HSV infection sites were inconsistently documented.

**Clinical Examination, Sampling, and Laboratory Procedures**. Standard proctologic examination included inspection, digital rectal examination, and anoscopy before and after intracanal application of 5% acetic acid. Two anal swabs were systematically collected for cytology (Dacron®, vortexed in PreservCyt® medium (Hologic, Boxborough, MA, USA) and HPV DNA testing (Minitip FLOQSwab® COPAN Diagnostics, CA, USA; placed in Universal Transport Medium [ UTM® COPAN Diagnostics, 3-mL, CA, USA]). Abnormal findings during anoscopy prompted targeted biopsies, fixed in 4 % formalin (Formol 4%, ALPHAPATH, Mudaison, France), and paraffin-embedded.

HPV DNA testing used the Cobas® 6800 system (Roche Diagnostics France, Meylan, France), detecting 14 HR-HPV genotypes including HPV16 and HPV18.

Cytology was processed with ThinPrep™ (HOLOGIC®, Malborough, MA, USA) with Papanicolaou staining and classified per 2014 Bethesda criteria: Negative for Intraepithelial Lesion Malignancy (NILM); Atypical squamous cells of undetermined significance (ASC-US); Low-Grade Squamous Intraepithelial Lesion (LSIL); Atypical squamous cells cannot exclude HSIL (ASC-H); High-Grade Squamous Intraepithelial Lesion (HSIL); and uninterpretable. Cytological abnormalities were defined as ASC-US+, comprising ASC-US, LSIL, HSIL, and ASC-H.

Biopsies were assessed with haematoxylin-eosin; p16 immunohistochemistry, with or without Ki-67, and graded as Anal Intraepithelial Neoplasia (AIN) 1, 2, or 3.

**Statistical Analysis**. Quantitative variables were expressed as median and inter quartile range (IQR) and qualitative variables as count and percentage, excluding missing values. Associations with HPV16 were estimated using a logistic regression model, assessed by likelihood ratio test and reported as adjusted odds ratio (OR) with 95% confidence interval (CI). A p-value <0.05 was considered significant. Analyses were performed with R v 4.4.3.

**Ethical Approval**.

Informed consent was waived by the HCL Ethics Committee (approval no. 24_5447) due to the retrospective, non-interventional design. Data were anonymized in accordance with the MR-004 framework and data protection regulations.

## 3 Results

**Fig. 1:**
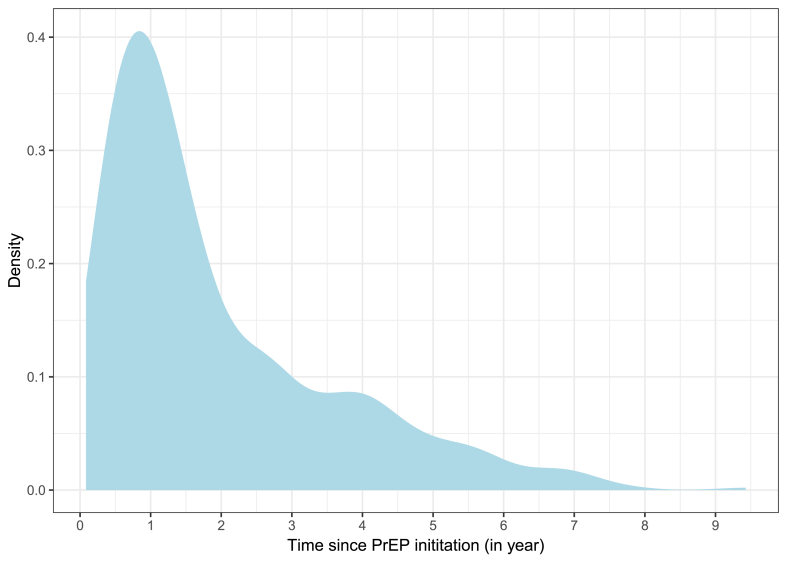
Distribution of participants according to years of PrEP use.

*Characteristics of the included population.* A total of 503 patients were included, 99.0% (n = 498) were males and 1.0% (n = 5) were transgender women; the median age at proctologic consultation was 38 [IQR, 31-47] years. The median duration of PrEP use was 476 [IQR: 277-1003] days (Fig. 1). Most participants lived in urban area ≥2000 inhabitants (96.6%, n = 482), with 69.1% (n = 345) in cities ≥100 000 inhabitants. A total of 26.2% (n = 132) were current or former smokers, 33.2% (n = 167) used novel psychoactive substances, 24.9% (n = 125) reported chemsex practices, and 17.1% (n = 86) received HPV vaccination prior to anal sampling for HPV. At least one previously PCR-confirmed CT and/or NG infection since PrEP initiation was documented in 56.5% (n = 284) of patients, while 24.7% (n = 124) were tested positive for CT and/or NG within the preceding year (Table 1).

**Table 1:** Demographic and clinical characteristics of participants included in the study.

	N = 503
Sex	
Male	498 (99·0)
Transgender Male to Female	5 (1·0)
Age, in years	38 (31-47)
< 30 years	113 (23)
30-50 years	303 (60)
> 50 years	87 (17)
Place of residence (department)	
Rhône	451 (90·4)
Ain	16 (3·2)
Isère	11 (2·2)
Others	21 (0·04)
Unknown	4 (0·01)
Place of residence (inhabitants)	
<2000	17 (3·4)
2000-20 000	74 (14·8)
20 000 – 100 000	63 (12·6)
> 100 000	345 (69·1)
Duration of PrEP use (in days)	476 (277-1003)
PCR HPV16	
Positive	149 (32·5)^a^
Negative	309 (67·5)^a^
Uninterpretable	45 (8·9)
Chemsex practice	125 (24·9)
NPS use	167 (33·2)
Current or former smoker	132 (26·2)
STI (CT and/or NG) in the previous 12 months (n = 403)	124 (24·7)
STI (CT and/or NG) since PrEP initiation	284 (56·5)
Cytology	
NILM	351 (69·8)
ASC-US	55 (10·9)
LSIL	44 (8·7)
ASC-H	4 (0·8)
Uninterpretable	49 (9·7)
Histologic sample available^b^	92 (20.1)
Histology	
AIN1	19 (3·8)
AIN2	5 (1·0)
AIN3	2 (0·4)
Condylomata acuminata	71 (14·1)
Hyperkeratosis	2 (0·4)
Molluscum	1 (0·2)
HPV vaccination^c^	86 (17·1)

Values are expressed as median (IQR) or n (%).

Abbreviations: PrEP, Pre-Exposure Prophylaxis; HPV, Human Papillomavirus; PCR, Polymerase Chain Reaction; NPS, New Psychoactive Substances; STI, Sexually Transmitted Infection; CT, *Chlamydia trachomati*s; NG, *Neisseria gonorrhoeae*; NILM, Negative for Intraepithelial Lesion or Malignancy; ASC-US, Atypical Squamous Cells of Undetermined Significance; LSIL, Low-Grade Squamous Intraepithelial Lesion; ASC-H, Atypical Squamous Cells, Cannot Exclude High-Grade Squamous Intraepithelial Lesion; AIN1, Anal Intraepithelial Neoplasia Grade 1; AIN2, Anal Intraepithelial Neoplasia Grade 2; AIN3, Anal Intraepithelial Neoplasia Grade 3; IQR, interquartile range.

a HPV PCR results were uninterpretable in 45/503 (8·9%) samples; percentages are calculated among interpretable samples (n = 458).

b Histologic assessment was only considered for participants with interpretable HPV PCR results (n = 458).

c Received at least one dose of an HPV vaccine.

Density plot showing the distribution of participants according to the duration of pre-exposure prophylaxis (PrEP) use, expressed in years. The median duration was 1.3 years (interquartile range [IQR], 0.8–2.7 years).

### 3.1 HPV 16 prevalence and its associated factors

PCR testing for HPV16 was performed on all the 503 anal samples, with 8.9% (n = 45) excluded due to uninterpretable results. Among the 458 interpretable samples, 32.5% (n = 149) were HPV16 positive.

Determinants of HPV16 infection were chemsex practices (OR = 1.76; 95% CI [1.14–2.71]; p = 0.011) and a history of anal CT and/or NG infection since PrEP initiation (OR = 1.64; 95% CI [1.10–2.47]; p = 0.016), or within the preceding year (OR = 1.61; 95% CI [1.04–2.50]; p = 0.033). In contrast, novel psychoactive substance use (OR = 1.33; 95% CI [0.89–2.00]; p = 0.167), history of smoking (OR = 1.24; 95% CI [0.80–1.91]; p = 0.33), age (OR = 0.997; 95% CI [0.979–1.02]; p = 0.758), duration of PrEP use (OR = 0.99; 95% CI [1]; p = 0.31), and HPV vaccination (OR = 1.21; 95% CI [0.71–2.03]; p = 0.481) were not associated with HPV16 infection. Similarly, place of residence—urban *versus* rural—was not associated with the likelihood of HPV16 infection (OR = 1.36; 95% CI [0.46–4.96]; p = 0.607), and no difference was observed upon stratification according to the size of the urban area (Table 2).

**Table 2:** Risk of Anal HPV16 Infection Associated with participant characteristics (Multivariable Logistic Regression).

Variable	Odds Ratio (OR)	95% Confidence Interval	p-value
Chemsex practice	1·76	1·14 – 2·71	**0**·**011**
Anal CT/NG infection in the past year	1·61	1·04 – 2·50	**0**·**033**
CT/NG infection since PrEP initiation	1·64	1·10 – 2·47	**0**·**016**
Use of novel psychoactive substances (NPS)	1·33	0·89 – 2·00	0·167
Smoking (current or past)	1.24	0·80 – 1·91	0·33
Age (continuous, per year increase)	0·997	0·979 – 1·02	0·758
Duration of PrEP use (days)	0·99	1 – 1	0·31
HPV vaccination (≥1 dose)	1·21	0.71 – 2.03	0·481
Urban *vs* rural residence	1·36	0·46 – 4·96	0·607

Abbreviations: CT, *Chlamydia trachomatis*; NG, *Neisseria gonorrhoeae*; PrEP, Pre-Exposure Prophylaxis; NPS, New Psychoactive Substances; HPV, Human Papillomavirus.

See *Methods* for the definition of urban and rural residence.

### 3.2 Cytological and histological findings

A cytological analysis was performed in all 458 HPV16-positive samples, with 44 (9.6%) samples deemed insufficient for interpretation due to insufficient nucleated squamous cellularity, yielding 414 valid results. Most were NILM (69.7%; n = 319/458) followed by ASC-US (11.1%; n = 51), LSIL (8.7%; n = 40), and ASC-H (0.9%; n = 4); no HSIL was detected. Overall, ASC-US+ were found in 22.9% (n = 95/414) of interpretable samples; higher prevalence was found in HPV16-positive patients (31.1%, n = 41/132) compared to HPV16-negative (19.1%, n = 54/282; OR = 1.90; 95% CI [1.18, 3.05]; p = 0.008).

Smoking and one-dose HPV vaccination were not associated with ASC-US + prevalence (OR = 1.23; 95%CI [0.73, 2.04]; p = 0.44 and OR = 0.95; 95%CI [0.51, 1.70]; p = 0.86, respectively; Table 3).

**Table 3:** Risk of cytological and histological abnormality associated with anal HPV16 infection and smoking (Multivariable Logistic Regression).

	ASC-US+^a^ cytological abnormality			AIN2+^b^ histological lesion		
	OR	95% CI	p value	OR	95% CI	p value
HPV16 infection	**1**·**90**	**1**·**18**–**3**·**05**	**0**·**008**	0·41	0·021 – 2·57	0·42
Smoking (current or past)	1·23	0·73 – 2·04	0·44	0·47	0·03 – 2·78	0·48
HPV vaccination (one dose)	0·95	0·51 – 1·70	0·85	—	—	—

Abbreviations: HPV, Human Papillomavirus; NILM, Negative for Intraepithelial Lesion or Malignancy; ASC-US, Atypical Squamous Cells of Undetermined Significance; LSIL, Low-Grade Squamous Intraepithelial Lesion; HSIL, High-grade Squamous Intraepithelial Lesion; ASC-H, Atypical Squamous Cells, Cannot Exclude High-Grade Squamous Intraepithelial Lesion; AIN1, Anal Intraepithelial Neoplasia Grade 1; AIN2, Anal Intraepithelial Neoplasia Grade 2; AIN3, Anal Intraepithelial Neoplasia Grade 3; CI, Confidence Interval.

a ASC-US + defined as ASC-US, LSIL, HSIL or ASC-H.

b AIN2+ defined as AIN2 or AIN3.

Histological data was available in 92 (20.1%) of the 458 patients with interpretable HPV16 testing, whereas 366 (79.9%) did not undergo biopsy due to the absence of visible lesions on anoscopy. Among patients with a biopsy, low-grade lesions (AIN 1 or condyloma) were most frequent (92.3%, n = 85/92, 18.6% overall, n = 85/458). High-grade squamous intraepithelial lesions (AIN 2+, defined as AIN2 or AIN3) were detected in 6 patients (6.5%, n = 6/92; 1.3% overall, n = 6/458). AIN 2+ prevalence was numerically higher in HPV16-negative patients (8.6%, n = 5/58) compared to HPV16-positive (2.9%, n = 1/34), without statistical significance (OR = 0.41; 95% CI: 0.021–2.57; p = 0.42). Smoking was not associated with AIN2+ lesions (OR = 0.47; 95%CI [0.03, 2.78]; p = 0.48).

Condylomas were observed in 14.4% (n = 66/458) of patients, corresponding to 71.7% (n = 66/92) of available biopsies. Considering all HPV-related histological lesions (AIN1–3 or condylomas), overall prevalence was 19.9% (n = 91/458).

## 4 Discussion

In the present large population of HIV-negative MSM and transgender women receiving PrEP, anal HPV16 prevalence reached 32.5%, substantially exceeding most prior estimates in this population, and approaching levels historically reported in HIV-positive MSM, a population considered at the highest risk for HPV-associated anal disease [3]. In MSM living with HIV, a meta-analysis reported anal HPV16 prevalence ranging from 21.7% to 38.2%, with pooled estimates around 28.5% [17]. Previous European studies focusing on PrEP users reported lower prevalence, ranging from 16.4% to 24.3% [[14], [15], [16]], while meta-analyses showed a prevalence of 12.5-14% in HIV-negative MSM not receiving PrEP [[17], [18], [19]].

Nearly one-quarter reported chemsex, and most had a recent CT or NG infection. The chemsex rate (24.9%) is consistent with recent data in French PrEP-using MSM, supporting the representativeness of our cohort [20]. Herein, both chemsex and recent bacterial STI were independently associated with HPV16 infection. A key strength of the present study is the inclusion of long-term PrEP users with often several months of exposure, unlike studies assessing HPV16 at PrEP initiation. This may have captured the risk compensation phenomenon, characterised by reduced condom use and increased rates of NG and CT infections in early PrEP users [14,15]. The association between bacterial STIs and HPV16, already observed in young men and mirroring the well-documented association in the female cervix, likely involves determinants beyond the behavioural factors alone [21]. A recent taxonomic study suggested that local microbiological and immunological factors may contribute to HPV susceptibility and reduced viral clearance [12]. These findings may reflect pathogen-driven alterations in the anorectal microbiota, loss of protective commensals, and a sustained pro-inflammatory mucosal environment favouring HR-HPV acquisition and persistence. Altogether, the present findings indicate that PrEP users’ exposure patterns and infection rates resemble those of HIV-positive populations, suggesting HIV status alone should not determine anal cancer screening eligibility in MSM and transgender women.

Herein, cytological abnormalities were frequent, with over 20% of patients classified as ASC-US+. These abnormalities were more frequent in HPV16-positive patients, which is consistent with the strong oncogenic potential of this genotype. For comparison, ASC-US + rates reach 50% in HIV-positive MSM, while prevalence among HIV-negative MSM ranges from 18.5% to 39% often without specifying the PrEP status [18,22]. In addition, data from the present study showed that up to 20% of asymptomatic PrEP users may present HPV-related lesions during routine follow-up. The prevalence of AIN2+ observed herein (1.3% overall) was slightly lower than commonly reported in HIV-positive MSM. Previous histological studies on HIV-positive MSM, using similar non-high resolution anoscopy (HRA) guided biopsy methods (i.e., sampling only visible lesions during standard anoscopy), have reported AIN2+ prevalence ranging from 3.3% to 6.7% [17,22,23]. To our knowledge, only one study directly compared HIV-positive and HIV-negative MSM without HRA, and found substantially higher rates (26% and 20.9%, respectively) [24]. In the present study, the limited number of biopsies —further reduced by the exclusion of samples from subsequent HRA visits for abnormal cytology—likely reduced the statistical power and may partly explain the absence of the significant association between HPV16 and high-grade histological lesions previously demonstrated [4,5].

Persistent HPV16 infection has been previously reported to amplify cumulative cancer risk [25]. In a meta-analysis, Wei et al. found that HIV-positive MSM had a lower HPV16 clearance than HIV-negative MSM (61 *vs.* 95 per 1000 person/months) [26]. Some PrEP users, although immunocompetent, could face risks similar to those of HIV-positive MSM, considering that the HPV16 clearance rate found by Cotte et al. in HIV-negative MSM using PrEP approaches the rate found by Wei et al. in HIV-positive MSM (12.6 per 1000 person/months) [16,26]. This clearance, combined with high infection rates, may lead to greater cumulative oncogenic risk over time, particularly in older patients or in those engaging in frequent receptive anal intercourses [16,26,27]—behaviours associated with increased mucosal trauma and reinfection [26]. The present cross-sectional design precluded direct assessment of persistence, but these factors warrant close surveillance/monitoring in the future.

In the present study, smoking was not associated with HPV16 infection and cytological abnormalities, unlike meta-analyses. This may reflect the absence of distinction between current and former smokers, as smoking cessation may reduce the HPV-related risk [28]. The small number of high-grade lesions precluded analysis of smoking and anal cancer risk, unlike previous studies suggesting smoking hinders lesion clearance and cause cytotoxic mucosal damage [29].

The present study found no association between vaccination and HPV16 infection among MSM and transgender women, unlike the meta-analysis by Wei et *al*. which reported benefits mainly in HPV-naïve individuals under 26 years —a feature that may not have been met herein [30]. The vaccination rate at proctology examination was consistent with the 2019 French National Authority for Health guidelines [43], but remained well below the 37% rate reported in a predominantly US-based meta-analysis [31,32].

The interpretation of the data from the present study is limited by the cross-sectional design, the absence of an HIV-positive control group, and the use of a single HPV test per participant, which precluded assessment of HPV16 clearance. Sex- and gender-based analyses were limited by the small number of transgender women included in the study. Since syphilis and anal HSV data were not collected, their potential contribution to mucosal susceptibility and HPV16 acquisition or persistence could not be assessed. Real-world inclusion criteria likely excluded the most severe anal HPV cases, potentially underestimating the true HPV16 burden. Nevertheless, the large sample size of the study — at least double previous similar studies — and extended recruitment period enhance robustness and generalisability.

From a public health perspective, these findings support reconsideration of current screening guidelines in favour of a targeted screening in high-risk PrEP users. A risk-adapted strategy—such as self-collected anal HPV16 testing followed by targeted proctology consultation for positive cases—could be feasible and acceptable, mirroring current practices in HIV-positive MSM. This approach could prioritize older individuals and those with documented bacterial STIs or high-risk sexual behaviours. Future studies should focus on the longitudinal evaluation of HPV16 persistence and clearance in PrEP users, as well as the incidence of high-grade anal intraepithelial lesions in this population. Prospective studies integrating repeated HPV testing, cytology, and high-resolution anoscopy would help refine risk stratification and determine optimal screening intervals. In addition, interventional approaches, including targeted HPV vaccination strategies in sexually active adults and behavioural risk reduction interventions, should be explored to reduce HPV acquisition and persistence. Finally, cost-effectiveness analyses are needed to assess the feasibility of implementing risk-adapted anal cancer screening strategies in HIV-negative MSM and transgender women receiving PrEP.

## 5 Conclusion

HPV16 prevalence is high in HIV-negative MSM and transgender women on PrEP, driven by high-risk behaviours and associated with cytological abnormalities. These findings support reconsidering anal cancer prevention and screening strategies beyond HIV-positive MSM and provide a basis for larger and prospective studies.

## CRediT authorship contribution statement

**Marion de Quillacq:** Writing – review & editing, Writing – original draft, Software, Investigation, Data curation. **Maxime Bonjour:** Writing – original draft, Visualization, Formal analysis. **Sarah Soueges:** Writing – original draft, Investigation. **Florent Valour:** Writing – review & editing, Writing – original draft, Investigation, Conceptualization. **Anne-Sophie Batalla:** Writing – review & editing, Investigation. **Florence Ader:** Writing – review & editing. **Hélène Lardot:** Investigation. **Anne Conrad:** Writing – review & editing. **Sylvie Radenne:** Writing – review & editing, Writing – original draft, Validation, Resources, Investigation. **Matthieu Godinot:** Writing – review & editing, Writing – original draft, Validation, Supervision, Methodology, Conceptualization.

## Data availability statement

The data that support the findings of this study are available on request from the corresponding author. The data are not publicly available due to privacy or ethical restrictions.

All authors have read and agreed to the published version of the manuscript.

## Preprint

This manuscript has not been posted on a preprint server.

## Writing assistance

No professional writing assistance was used in the preparation of this manuscript.

## Declaration of generative AI and AI-assisted technologies in the manuscript preparation process

During the preparation of this work, the author(s) used AI tools to assist in the creation of the graphical abstract.

## Funding

This research did not receive any specific grant from funding agencies in the public, commercial, or not-for-profit sectors.

## Declaration of competing interest

The authors declare that they have no known competing financial interests or personal relationships that could have appeared to influence the work reported in this paper.
